# No Evidence of Moderated Impulsivity Following Administration of the IMPase Inhibitor Ebselen in Healthy Adults

**DOI:** 10.1002/hup.70040

**Published:** 2026-04-21

**Authors:** Matthew P. G. John, Luke Earnshaw, Nikhil Gauri Shankar, Jawad Sultan Raja, Timothy J. Davies, Phillip J. Cowen, Trevor Sharp, Alberto Salmoiraghi, Robert D. Rogers

**Affiliations:** ^1^ School of Psychology and Sports Science Bangor University Bangor Gwynedd UK; ^2^ Mental Health and Learning Disability Division Ysbyty Gwynedd, Penrhosgarnedd Bangor UK; ^3^ Department of Psychiatry Warneford Hospital University of Oxford Oxford UK; ^4^ Oxford Health NHS Foundation Trust Oxford UK; ^5^ Department of Pharmacology University of Oxford Oxford UK

**Keywords:** ebselen, impulsivity, inositol monophosphatase, myo‐inositol

## Abstract

**Objective:**

Impulsivity is a transdiagnostic risk factor for numerous health morbidities and is strongly associated with early relapse and poor treatment outcomes in addictions and mood‐disorders. Lithium carbonate can be helpful in moderating the impulsive behaviors associated with mania, possibly mediated by reduced *myo*‐inositol activity following inhibition of the enzyme inositol monophosphatase (IMPase). We tested the hypothesis that impulsivity—as motor disinhibition, decisions without adequate information, and stronger preferences for small immediate rewards over larger later rewards—can be moderated by the IMPase inhibitor ebselen in healthy adult volunteers.

**Methods:**

One hundred and thirty healthy adults completed a between‐subjects, double‐blind, placebo‐controlled protocol. Over 2 days, participants received a previously validated dose of 1800 mg of ebselen or placebo before completing tests of impulsivity and decision‐making.

**Results:**

There were no substantive changes in any measure of impulsivity following treatment with ebselen compared with placebo. Neither was there any convincing evidence of stronger treatment effects in high‐trait impulsive participants compared with low‐trait participants.

**Conclusion:**

These results fail to replicate findings that ebselen administration moderates validated measures of impulsivity in healthy adults, at least at doses shown to reduce *myo*‐inositol within the medial prefrontal cortex and produce changes in emotional processing and reward‐based learning.

**Abbreviations**
CPT: Continuous Performance Test
FERT: Face Emotion Recognition Task
IMPase: inositol monophosphatase
IST: Information Sampling Task
MCQ: Monetary Choice Questionnaire
SST: Stop‐Signal Task
TA: Titrating Alternatives

## 1 Introduction

Impulsivity, expressed as rapid actions or decisions producing harmful effects, is a salient feature of the behavioral phenotypes of various psychiatric illnesses, including attention‐deficit/hyperactivity disorder, alcohol and substance use disorders, certain personality and conduct disorders, and disordered gambling and other behavioral addictions (Kirby et al. 1999; Levitt et al. 2020; Madden et al. 2011; Simon et al. 2001). Heightened impulsivity is associated with earlier onset of these illnesses, increased risk of early relapse, suicidality, and poorer treatment outcomes (Adinoff et al. 2016; Liu et al. 2017; Sliedrecht et al. 2021). This broad transdiagnostic involvement highlights the potential value of moderating impulsivity as a treatment strategy (Kozak et al. 2019).

Current pharmacotherapies show inconsistent efficacy in moderating impulsivity, depending on the clinical population and outcome measure (Anderson et al. 2021; Felthous et al. 2021; Grassi et al. 2021; Kozak et al. 2019; Schwartz et al. 2023). However, lithium carbonate is consistently effective in reducing the impulsivity characteristic of manic states (McKnight et al. 2019) and in moderating broader patterns of self‐harm, suicidality, and impulsive aggression in some patient groups (Cipriani et al. 2013; Felthous et al. 2021; R. M. Jones et al. 2011; Murphy et al. 2024; Nabi et al. 2022; Sheard et al. 1976; Simon et al. 2001). Small‐scale studies report reduced impulsive gambling following lithium treatment in individuals with co‐occurring bipolar spectrum disorder and disordered gambling (Hollander et al. 2005), possibly mediated by altered orbitofrontal‐cingulate activity (Hollander et al. 2008). Finally, in animal models, acute and chronic lithium can attenuate premature responding in the 3‐ and 5‐choice Serial Reaction Time Tasks (Adams et al. 2020; Ohmura et al. 2012); and, in humans, improve decision‐making in the Iowa Gambling Task (Adida et al. 2015).

Lithium's side‐effects profile (Atack 2000; McKnight et al. 2012) and narrow therapeutic index (Bortolozzi et al. 2024) limit its clinical use, but highlight the importance of identifying which of its pharmacological mechanisms (Gao and Calabrese 2021) mediate its putative anti‐impulsive effects. According to the inositol depletion hypothesis (Berridge et al. 1989), lithium's inhibitory actions on the enzyme inositol monophosphatase (IMPase) reduce *myo*‐inositol levels, thereby moderating phosphatidylinositol (PI) signaling (Allison et al. 1976; Alllison and Stewart 1971). Changes in PI signaling stabilise downstream monoamine release and postsynaptic receptor activity (Bermingham et al. 2016) to support broader cognitive and affective functions, including those implicated in impulse control (Dalley and Robbins 2017). In this way, the moderation of *myo*‐inositol availability offers a viable therapeutic target for addressing impulsivity in clinical groups.

Ebselen, a bioavailable antioxidant, is an effective IMPase inhibitor that reduces whole brain *myo*‐inositol concentrations in rodents (Singh et al. 2013) and within the medial prefrontal cortex in both healthy adults (Masaki et al. 2016b) and treatment‐resistant depressed patients (Ramli et al. 2024). The drug also shows promising results as an adjunctive treatment for mania (Sharpley et al. 2020). In animals, ebselen inhibits PI‐linked 5HT_2A_ receptor function in different models (Antoniadou et al. 2018), reduces premature responding in the 5‐Choice Serial Reaction Time Task, and blocks cocaine‐induced patterns of premature responding (Barkus et al. 2018). Small‐scale studies with healthy human volunteers show that short courses of ebselen can moderate rapid patterns of betting in the Cambridge Gambling Task (Masaki et al. 2016a), but also diminish sensitivity to rewarding outcomes in a probabilistic decision‐making task (Singh et al. 2016) and improve the recognition of positive emotions in the face (Masaki et al. 2016a; Singh et al. 2016). Collectively, these findings suggest that ebselen can moderate impulsive behaviors and the related reinforcement‐learning that support decision‐making between value‐laden alternatives.

Here, in a pre‐registered (between‐subjects) experiment, we sought to test the hypothesis that depleted *myo*‐inositol levels following administration of the IMPase inhibitor ebselen (1800 mg over 2‐day) can diminish impulsivity in a community sample of healthy (non‐clinical) adults. We operationalised impulsivity in three core forms: (i) ‘motor impulsivity’ as inhibitory control in the Stop‐Signal Task (Verbruggen et al. 2012) and Continuous Performance Test (Dougherty et al. 2002); (ii) ‘reflection impulsivity’ in the Information Sampling Task (Clark et al. 2006) and Observe‐or‐Bet Task (Navarro et al. 2016); and (iii) ‘delay discounting’ measured with the Titrating Alternatives Task (Du et al. 2002; Rung et al. 2018) and the Monetary Choice Questionnaire (Kirby et al. 1999).

Trait impulsivity was measured using the Barratt Impulsivity Scale (BIS‐11) (Patton et al. 1995; Stanford et al. 2009) to test the secondary hypothesis that IMPase inhibition following ebselen treatment will produce larger reductions in measured impulsivity in high‐trait impulsive individuals compared with low‐trait individuals, potentially increasing confidence in ebselen's therapeutic potential. Finally, to replicate observations of altered emotional processing following ebselen administration (Masaki et al. 2016a; Singh et al. 2016), we also assessed facial emotion recognition using the Face Emotion Recognition Task (FERT) (Harmer et al. 2004).

## 2 Methods

### 2.1 Participants

The study was given a favorable opinion by the National Health Service Research Ethics Committee (Wales REC 2) (IRAS ID: 244365). All participants provided voluntary and informed consent.

One‐hundred‐and‐thirty‐two participants (*N*_male_ = 66, *N*_female_ = 66) enrolled in the study. Exclusion criteria were as follows (i) current or historical DSM‐V Axis I psychiatric disorder, bipolar disorder, depressive illness or anxiety disorder, or substance misuse or dependence disorder assessed using the Non‐Patient Edition of the Structured Clinical Interview for Diagnostic and Statistical Manual for Mental Health Disorders (First et al. 2015); (ii) significant current illness (e.g., diabetes, hypertension, or cardiovascular illness); (iii) current use of regular prescription medication (apart from the contraceptive pill); (iv) pregnancy or lactation; (v) smoking more than 10 cigarettes per day; (vi) BMI less than 18 or greater than 34; (vii) participation in another study of an investigational drug within the previous 3 months; (viii) and non‐fluent English. Participants were asked to abstain from alcohol and to use a listed method of contraception during the study period.

Data collection began in December 2021 while Welsh Government‐mandated COVID‐19 restrictions were in place. Therefore, additional exclusion criteria included current symptoms of COVID‐19 (continuous cough, high temperature, loss of or change to sense of smell or taste) or ‘long’ COVID (e.g., persisting tiredness, shortness of breath, concentration problems, difficulties sleeping). All data were collected first between December 2021 and June 2022, and then, following revisions to the protocol (see below), between November 2022 and June 2024.

### 2.2 Design

The study consisted of a double‐blind, placebo‐controlled, gender‐balanced design. Originally, a 4‐day protocol included three arms: 60 participants receiving ebselen, 60 receiving placebo, and 60 participants receiving lithium carbonate as a positive comparator for ebselen's putative anti‐impulsive effects. However, the interruption of the COVID‐19 pandemic and recruitment challenges associated with the biochemical screening for the lithium and intrusive COVID‐19 restrictions made it necessary to drop the lithium arm and switch to a simpler 2‐day protocol with two sets of participants receiving ebselen or placebo.

The 4‐ and 2‐day protocols did not differ in the scheduling of doses across the protocol or the total dose of ebselen administered (see Supporting Information S1: Table S1); and the data of the 10 participants who received ebselen and the 8 who received placebo in the 4‐day protocol showed no substantial differences to those of the larger number of participants who completed the 2‐day protocol (Supporting Information S1: Table S2). Therefore, the data pooled across protocols are presented here. Two placebo participants (both female) withdrew from the study having started the study medications but prior to cognitive testing. In total, the analysis was completed over the data from 130 participants (*N*_ebselen_ = 66, *N*_placebo_ = 64).

Participants received 3 × 200 mg capsules of ebselen or placebo on 3 separate occasions over 2 days: on Day 1, at 9a.m. and 4p.m.; and on Day 2, at 10a.m., 2 h prior to completion of the cognitive assessments of impulsivity. Thus, the first and second doses were separated by 7 hours, and the second and third doses by 18 hours. This dosing schedule was chosen to replicate those that produce decrements in *myo*‐inositol levels within medial prefrontal cortex and have shown altered cognitive processing in healthy adults (Masaki et al. 2016a; Singh et al. 2016). The ebselen capsules did not contain any excipients and the placebo capsules consisted of microcrystalline cellulose.

All dosing was performed on‐site at Bangor University, under the supervision of one of the researchers (MPGJ or LE). Information about any side‐effects (headache, nausea, fatigue, thirst, frequent urination, stomach upset, and dizziness) was collected at the start of the second and third dosing visits.

### 2.3 Background, Mood/Affect and Personality Questionnaires

Educational history was specified using the International Standard Classification of Education (ISCED) (EurostatStatistics 2015). Baseline depression and anxiety symptoms were assessed with the Beck Depression Inventory (BDI) (Beck et al. 1996) and the trait form of the State and Trait Anxiety Inventory (STAI‐Y2) (Spielberger 1970). Trait impulsivity scores were collected with the Barratt Impulsivity Scale (BIS‐11) (Patton et al. 1995; Stanford et al. 2009). Mood elevation experiences—strongly associated with impulsivity in clinical and community populations (Gillett et al. 2021)—were assessed with the Mood Disorders Questionnaire (Hirschfeld 2002). Baseline mood and affect were assessed using the Befindlichskeit scale of mood and energy (BFS) (Von Zerssen et al. 1974) and the Profile of Mood States (POMS) (McNair et al. 1971). On the day of testing, participants completed the BFS and POMS for a second time, in addition to the state versions of the STAI (STAI‐Y1) and the Positive and Negative Affect Scale (PANAS‐S) (Watson et al. 1988).

### 2.4 Laboratory Assessments of Impulse Control

In addition to tests of motor impulsivity, reflection impulsivity, delay discounting, and emotional recognition (Harmer et al. 2004), the protocol included tasks tapping related cognitive and affective functions: (i) probability discounting (Du et al. 2002; Madden et al. 2009); (ii) risk‐based decision‐making (Rogers et al. 2003); (iii) resource management (Rauwolf et al. 2024); and (iv) delayed emotional memory (Harmer et al. 2004). The entire study dataset, along with a summary of the broadly null results from the additional cognitive tasks can be found at: https://osf.io/45xu9/.

### 2.5 Reflection Impulsivity

#### 2.5.1 Information Sampling Task (IST) (Clark et al. 2006 for Details)

The IST is a measure of reflection impulsivity, in which participants were required to decide how much information to gather before making decisions to maximise nominal experimenter‐defined rewards as 'points'. Participants viewed visual displays of gray boxes containing hidden colored (blue or yellow) squares and were invited to open as many or as few boxes as they wished before making a judgment about which color of square was predominant. The two principal dependent measures of the IST were: (i) the total number of boxes opened before making declarations; and (ii) *p(Correct*), the momentary probability of declaring the correct color. *p(Correct*) can be interpreted as an index of reflection impulsivity, with higher values indicating less reflection impulsivity than lower values (Bennett et al. 2017).

#### 2.5.2 Observe‐or‐Bet Task (Navarro et al. 2016)

The observe‐or‐bet task is a second measure of reflection impulsivity in which, over a series of trials, participants were invited to balance observations of a ‘blox machine’—a simple device in which red or blue lights were illuminated probabilistically—against bets of experimenter‐defined points on its hidden state: the machine's bias towards one of the two colors. Two key measures were: (i) the number of observations across all blocks, and (ii) the number of points earned.

### 2.6 Motor Impulsivity

#### 2.6.1 Stop‐Signal Task (SST) (Verbruggen et al. 2012, 2013)

The SST provides an assay of motor inhibition. In a discrete‐choice reaction time task, participants were asked to respond rapidly to a series of left or right arrows with the corresponding left or right index‐finger key‐presses; except on a subset of trials on which these stimuli were replaced by a ‘stop signal,’ requiring participants to inhibit the already‐activated response. Two key measures were assessed: (i) the probability of erroneous responses on stop‐signal trials, or *p(response | stop‐signal)*; and (ii) the stop‐signal response time itself (SSRT), an indirect measure of the average time (ms) needed to cancel a motor response, with smaller values indicating more efficient impulse control as motor inhibition than larger values.

#### 2.6.2 Continuous Performance Test (CPT) (Dougherty et al. 2002)

The Continuous Performance Test (CPT) is a working memory and sustained attention task, that requires the management of impulsive responses to maintain mental focus. Participants were shown briefly presented pairs of five‐digit numbers and were required to respond whenever the two numbers were identical (‘target’ trials) but not respond when they were different (‘catch’ and ‘filler’ trials). The principal outcome measures included (i) mean RTs to target trials and (ii) the ratio of responses to catch trials (‘commission errors’) to responses to target trials (‘correct detections’).

### 2.7 Delay Discounting

#### 2.7.1 Titrating Alternatives Discounting Task (Du et al. 2002; Rung et al. 2018)

Participants were asked to indicate their preferences between hypothetical sums of money available immediately and larger sums of money only available following varying delays. The delays to the greater sums of money increased across blocks of trials, while the value of the smaller sums was adjusted dynamically within blocks to converge on participants' indifference points. The TA discounting task yielded two indices of delay‐based impulsivity: (i) the fitted hyperbolic rate of discounting reward value as a function of delay, *k* (Mazur 2013); and (ii) the area under the curve (AUC; Myerson et al., 2001). The TA data were screened for non‐systematic patterns of preferences over the elicitation using established criteria (Johnson and Bickel 2008; Rung et al. 2018).

#### 2.7.2 The Monetary Choice Questionnaire (MCQ) (Kirby et al. 1999)

The MCQ is a questionnaire elicitation of individuals' hyperbolic rate of discounting reward value with delay, comparable to discrete‐choice elicitations such as the TA discounting task (Kaplan et al. 2016; Mazur 2013).

### 2.8 Emotion Recognition (Control) Task

#### 2.8.1 Face Emotion Recognition Task (FERT) (Harmer et al. 2004)

The FERT assesses recognition speed and accuracy of six emotional states in pseudo‐random series of visually presented faces (happiness, surprise, sadness, fear, anger, disgust, and neutral). Participants were asked to indicate which emotion was displayed by pressing one of seven labeled keys. Responses were untimed, but participants were instructed to answer as quickly and as accurately as possible.

### 2.9 Data Analysis

Our design, materials, and statistical approach were pre‐registered with AsPredicted.org for sample sizes of 60 participants in each of the ebselen and placebo groups (https://aspredicted.org/sjg9‐r62z.pdf). Inspection of Masaki et al. (2016a) suggested a Cohen's *d* of around 0.50 for a main effect of ebselen over placebo treatment. Assuming a moderate effect size (*f* ^2^ = 0.06) and using OLS regression over the primary outcome measures with sample sizes of 66 ebselen and 64 placebo participants of the adjusted design, we estimated an implied statistical power of 0.88 to detect main effects of treatment at *p* < 0.05 (one‐tailed). Assuming smaller effect sizes for the moderation of treatment effects by the BIS‐11 scores for trait impulsivity (*f* ^2^ = 0.04), the estimated power to detect interaction effects dropped to 0.74 at *p* < 0.05 (one‐tailed). All of the study data, as well as our models in R (R Studio version 4.3.3; https://posit.co/), can be found at https://osf.io/45xu9/.

Main effects were tested with linear regression models that included treatment group (ebselen vs. placebo)—with the latter as the referent—and trait impulsivity (BIS‐11 scores) (Patton et al. 1995) as predictors. Next, the moderation of treatment effects by trait impulsivity was tested using full models that included the added interaction terms. On the basis of previous reports of positive associations between measures of impulsivity and BIS‐11 subscale scores, interaction effects on the SST and CPT were tested in relation to the motor impulsivity subscale of the BIS‐11 (Caswell et al. 2015) while interaction effects on the IST, the Observe‐Bet task, the TA, and the MCQ were tested with the non‐planning subscale (Brynte et al. 2023; De Wit et al. 2007; Mobini et al. 2007).

All statistical analysis related to a priori predictions. However, to achieve some control over multiple comparisons and the risk of Type I errors, we confined our statistical tests to two principal outcome measures per task. To ensure that results were not unduly dependent on outliers, the models were re‐run following removal of high‐influence datapoints (defined by a Cook's distance equal to or greater than 4/*n*). In general, the impact of high‐influence datapoints was negligible (see Supporting Information S1: Table S3). *k* values were log‐transformed prior to analysis.

## 3 Results

### 3.1 Group Matching

The ebselen and placebo participants were closely matched in terms of demographic characteristics (see Table 1). The mean ages (*t* (128) = 0.42, *p =* 0.67), gender balance (χ^2^ (1) < 0.01, *p =* 1.00), and educational histories (χ^2^ (4) = 3.09, *p =* 0.54; see Supporting Information S1: Table S4) were closely comparable across the two treatment groups. Similarly, the groups did not differ markedly in terms of mood disturbance, recent depressive symptoms, or trait impulsivity (all *t* (128) ≤ |0.59|, *p = *> 0.55).

**TABLE 1:** Age, genders and mean (± standard errors) baseline trait and state psychometric scores.

	Ebselen	Placebo
Demographic and trait characteristics		
Age	31.65 ± 1.56	30.81 ± 1.57
Gender (male: female)	33:33	33:31
State‐trait affect inventory—Y2	35.97 ± 1.00	35.47 ± 1.13
Befindlischkeit	9.15 ± 1.05	8.28 ± 1.04
Mood disorders questionnaire	5.82 ± 0.47	5.59 ± 0.47
Beck depression inventory ‐ II	6.12 ± 0.68	5.71 ± 0.68
Profile of mood states	2.46 ± 2.15	1.41 ± 2.22
Barratt impulsivity scale 11 (total)	59.36 ± 1.37	59.25 ± 1.35
BIS 11 (attentional)	14.80 ± 0.51	15.40 ± 0.54
BIS 11 (motor)	22.40 ± 0.50	21.50 ± 0.48
BIS 11 (non‐planning)	21.80 ± 0.62	22.70 ± 0.60
State mood and affect at test		
Befindlischkeit	7.20 ± 1.00	6.69 ± 0.88
State‐trait affect inventory—Y1	28.64 ± 0.81	29.33 ± 0.94
Profile of mood states	−0.23 ± 2.07	−1.19 ± 2.46
Positive and negative affect scale +	34.39 ± 0.99	34.97 ± 0.85
Positive and negative affect scale −	11.21 ± 0.25	11.48 ± 0.40

#### 3.1.1 Side‐Effects and Treatment‐Related Changes in Affective State

The incidence of side‐effects—headaches, stomach upset, nausea, fatigue, frequent urination, thirst, dizziness—were comparable between the participant groups, with marginal reductions in moderate or severe effects following ebselen compared with placebo (all *χ* ^2^ ≤ |3.68|, *p = *> 0.31; see Supporting Information S1: Table S5). At test, mood was not markedly different following treatment with ebselen compared to placebo as measured with the BFS (see Table 1; *t* (128) = 0.38, *p =* 0.70) and the POMS (*t* (128) = 0.30, *p* = 0.76). State anxiety, state positive affect, and state negative affect were also broadly equivalent between the two groups (all *t* (128) ≤ |0.58|, *p = *> 0.53).

#### 3.1.2 Laboratory Measure of Reflection Impulsivity, Motor Impulsivity and Delay‐Based Impulsivity

As a first sense check, we compared the main outcome measures for each of the IST, SST, CPT, TA discounting task, and MCQ from our participant sample (pooled over the ebselen and placebo groups) with the values reported previously from healthy, non‐clinical populations. Overall, our data are comparable with prior research (see Supporting Information S1: Table S6).

### 3.2 Reflection Impulsivity

**FIGURE 1:**
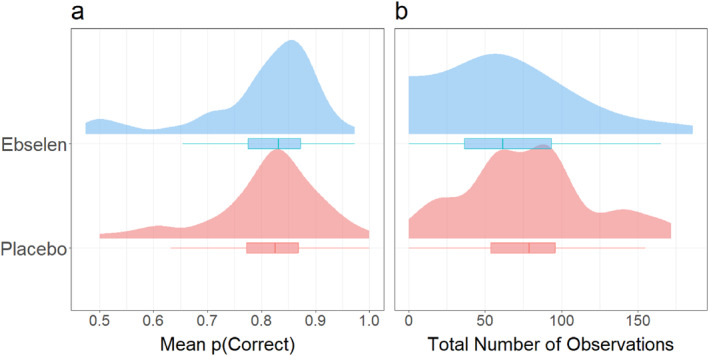
Distributions, means and interquartile ranges of mean *p* (Correct) in the Information Sampling Task (IST) (Clark et al. 2006) (a) and total number of observations across games in the Observe‐or‐Bet task (Navarro et al. 2016) (b).

Looking at the distribution of scores for the IST, the ebselen participants opened marginally fewer boxes than the placebo participants before declaring a majority color (5.61 ± 0.41 vs. 5.97 ± 0.48, *β* = −0.36 ± 0.63) and showed almost identical momentary probabilities of declaring the correct color during the task, *p(Correct)* (see Figure 1a; *β* = −0.01 ± 0.02).

On the Observe‐or‐Bet task, there were no marked differences between the placebo and ebselen participants' mean number of observations of the ‘blox’ machines (see Figure 1b; *β =* −11.09 ± 7.53) or mean number of points earned (41.90 ± 3.52 vs. 42.70 ± 3.73, *β =* 0.78 ± 5.26).

### 3.3 Motor Impulsivity

**FIGURE 2:**
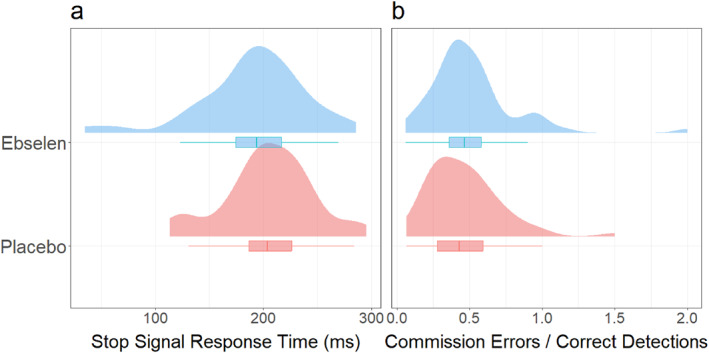
Distributions, means and interquartile ranges of stop signal times in the Stop‐Signal Task (SST) (Verbruggen et al. 2012) (a) and ratio of commission errors to correct detections in the Continuous Performance Test (CPT) (Dougherty et al. 2002) (b).

Stop‐signal data were collected for 92 participants (*N*_placebo_ = 48, *N*_ebselen_ = 44). Critically, the SSRT did not differ between the ebselen and placebo participants (see Figure 2a, *β =* −9.69 ± 10.81). The *p(response | stop‐signal)* was negligibly greater among ebselen than among placebo participants (*β* = 0.02 ± 0.01). The ratio of commission errors to correct detections on the CPT was marginally but not significantly greater among ebselen participants compared with placebo participants (see Figure 2b; *β =* 0.05 ± 0.05), while RTs on target trials were fractionally faster (446.00 ± 2.80 ms vs. 441.00 ± 3.17 ms, *β* = −5.00 ± 4.22).

### 3.4 Delay Discounting

**FIGURE 3:**
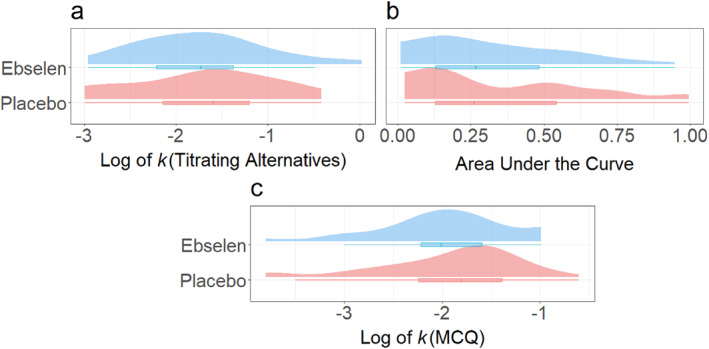
Distributions of delay discounting rates (represented as log k values and area‐under‐the‐curve; AUCs), means and interquartile ranges from the Titrating Alternatives elicitation (Rung et al. 2018) (a, b) and the Monetary Choice Questionnaire (Kirby et al. 1999) (c).

Following Johnson and Bickel (2008) and Rung et al. (2018), the data of 6 placebo and two ebselen participants were identified as non‐systematic in the TA task. However, omitting these values from the models did not substantially change the overall pattern of indifference points or the statistics of the inter‐group tests (see Supporting Information S1: Table S7). Therefore, these participants’ data were retained. Overall, there were no differences in the mean log *k* and AUC values of the ebselen participants compared with the placebo participants (see Figure 3a, *β* = −0.03 ± 0.12, and Figure 3b, *β* = −0.04 ± 0.04, respectively). For the MCQ, there was little evidence of either gentler or steeper discounting of delayed rewards among participants given ebselen compared with placebo (see Figure 3c; *β =* −0.28 ± 0.26).

#### 3.4.1 Moderation of Treatment Effects by Trait Impulsivity

Pooling across treatment groups, correlation coefficients between the task‐based measures of impulsivity and BIS‐11 scores were weak to modest (see Supporting Information S1: Figure S1). Participants with patterns of rapid delay discounting on the TA task tended to have higher BIS‐11 non‐planning scores, higher BIS‐11 motor impulsivity scores, and higher BIS‐11 total scores (−0.26 = < *r* < = −0.24, *p*s < 0.01). Correlations among the task‐based reflection, motor, and delay‐dependent impulsivity measures were also moderate at best. However, participants who scored the most points on the Observe‐or‐Bet task tended to report low log *k* values from the TA task and MCQ (*r* = −0.24, *p* < 0.01 and *r* = − 22, *p* < 0.01). Similarly, participants who reported the highest proportion of correct color judgments on the IST showed lower log *k* values on the MCQ (*r* = −0.20, *p* < 0.05).

There was no evidence for stronger or weaker treatment effects of ebselen on the principal measures of reflection impulsivity among individuals with higher compared with lower trait non‐planning impulsivity scores on the BIS‐11 (all *β*s < = |0.82| ± 1.08). Similarly, treatment effects of ebselen were broadly equivalent across behavioral measurements of motor impulsivity among individuals reporting low and high trait motor impulsivity (all *β*s ≤ |3.57| ± 15.91). However, the data showed complex but untypical patterns of delay discounting as a function of BIS‐11 non‐planning scores as described below.

Ebselen administration was associated with marginally lower log *k* values relative to placebo among participants with lower non‐planning impulsivity scores (indicating gentler discounting of delays to future rewards), but higher log *k* values among participants with higher non‐planning scores (indicating steeper discounting) (see Supporting Information S1: Figure S2a; *β* = 0.08 ± 0.02, *t* (124) = 3.45, *p* = 0.001). The AUC data (in which lower values indicate steeper discounting rates) showed the complement of this pattern (Supporting Information S1: Figure S2b; *β* = −0.02 ± 0.01, *t* (124) = −2.49, *p* = 0.01): ebselen treatment was linked to higher AUC values relative to placebo among participants with lower non‐planning impulsivity scores (indicating gentler delay discounting rates), but lower AUC values among participants with higher non‐planning scores (indicating more rapid discounting).

Finally, for the MCQ data, ebselen was associated with marginally lower log *k* values (and gentler discounting of delays to future rewards) compared with placebo among participants with low non‐planning BIS‐11 scores, but higher log *k* values (and steeper discounting rates) among participants with higher non‐planning scores (see Figure 2c *; β =* 0.11 ± 0.05, *t* (125) = 1.99, *p =* 0.05).

### 3.5 Face Emotion Recognition Task (FERT)

Two ebselen participants failed to correctly identify any emotional expressions from the FERT and were excluded. Ebselen produced no appreciable changes in the percentages of correctly identified positive expressions (happy + surprised; 75.60 ± 1.08 vs. 75.40 ± 0.75; *β* = −0.17 ± 1.28) or negative expressions (sad + fear + disgust + anger; 48.20 ± 1.09 vs. 49.60 ± 1.01; *β* = 1.41 ± 1.47). Participants' times to identify positive and negative emotions were also unchanged (1753 ± 48 ms vs. 1645 ± 49 ms, *β* = 107.29 ± 68.27; and 1897 ± 47 ms vs. 1867 ± 56 ms, *β =* 30.4 ± 72.26, respectively).

## 4 Discussion

These results show, in contrast to our predictions, no substantial changes in laboratory assessments of impulsivity following administration of ebselen compared with placebo in healthy adults. The distributions and mean scores on tests of motor impulsivity, reflection impulsivity, and delay discounting in community‐recruited healthy adults treated with 1800 mg ebselen over 2 days were indistinguishable from gender‐ and age‐matched adults treated with placebo. There was also no substantive evidence that patterns of impulsive behavior were altered to a greater extent in high trait impulsive individuals compared with low trait impulsive individuals. These findings suggest that ebselen administration does not markedly moderate any of the principal expressions of impulsivity at a dose previously shown to decrease *myo*‐inositol levels within the medial prefrontal cortex in both healthy adults (Singh et al. 2016) and in treatment‐resistant depressed patients (Ramli et al. 2024), and to moderate both reward‐based decision‐making and emotional processing (Singh et al. 2016).

The design used here had a number of strengths. First, the study was comparatively well‐powered with a sample size of 130 participants, offering an implied power of 88% to detect treatment differences at an estimated effect size of *f* ^2^ = 0.06. Second, our laboratory assessments afforded two dependent measures for each of motor impulsivity, reflection impulsivity, and delay discounting, which (with the single exception of the Observe‐or‐Bet task) have been used successfully in previous studies of impulsivity, its psychopharmacology, or associations with clinical disorders and risk factors (Hıdıroğlu et al. 2015; Lawrence et al. 2009; Moody et al. 2016; Raeder et al. 2025; Swann et al. 2005). Thus, it is unlikely that the null findings reported here can be accounted for by the use of unvalidated or insensitive measures. Third, in the context of previous investigations, the patterns evident in the performance of our participants across these tasks (see Supporting Information S1: Table S6) were comparable to those reported previously in comparable samples of healthy control adults (Bailey et al. 2018; Boyd et al. 2024; Brudan et al. 2024; Clark et al. 2006; Halilova et al. 2024; Kirenskaya et al. 2021; Todesco et al. 2025; Verbruggen et al. 2013), minimising the likelihood of ceiling effects militating against the detection of treatments effects.

As with all failures to replicate, caveats apply. The behavioral effects of ebselen in preclinical models appear to be dose‐dependent (Antoniadou et al. 2018; Barkus et al. 2018; Singh et al. 2013), and the single report of diminished impulsive responding following ebselen administration in human subjects delivered total doses of 3600 mg over 2 days (Masaki et al. 2016a). This is twice the dose used here over the same interval, raising the possibility that the absence of treatment effects in our data reflects an insufficient dose. However, reductions in cortical *myo*‐inositol within the medial prefrontal cortex are evident at doses of 1800 mg in both healthy adults (Singh et al. 2016) and in treatment‐resistant depressed patients (Ramli et al. 2024). This dose also produces changes in reward‐based learning and emotional processing (Singh et al. 2016), making it unlikely that the 1800 mg dose used here had no central actions on IMPase and *myo*‐inositol levels. Further, in a clinical context, ebselen has shown efficacy in treating noise‐induced hearing loss at doses of 400 mg (taken twice daily over 4 days), but not at doses of 200 mg or 600 mg (Kil et al. 2017). Thus, while acknowledging that higher doses of ebselen may show stronger anti‐impulsive effects than found here with doses of 1800 mg, it is possible that our findings reflect a more complex non‐monotonic dose‐response relationship.

Finally, we found no evidence of a positive bias in the recognition of facial emotional expressions using the FERT (Harmer et al. 2004) following ebselen compared with placebo. However, previous findings in relation to emotion recognition are inconsistent across studies. While both Masaki et al. (2016a) with a total dose of 3600 mg, and Singh et al. (2016) with a total dose of 1800 mg, reported increased accuracy for the classification of happy and surprised faces in ebselen‐treated participants compared with placebo‐treated participants (with the latter experiment also reporting greater accuracy for disgusted faces), Ramli et al. (2024) found no benefits of ebselen on the recognition of any emotion in a sample of participants with treatment‐resistant depression. Collectively, the previous and these present findings point to variable or at least dose‐dependent patterns of cognitive‐affective changes following ebselen treatment.

Varying expressions of impulsivity are mediated by the activity of interacting but dissociable monoamine and glutamate systems (Chernoff et al. 2024; da Cunha‐Bang and Knudsen 2021; Dalley and Robbins 2017; J. A. Jones et al. 2021; Toschi et al. 2021). Perhaps unsurprisingly, associations between behavioral, performance‐based, and self‐report measures of impulsivity (Caswell et al. 2015; MacKillop et al. 2016; Stahl et al. 2014), including those of the BIS‐11 and the SST, are modest at best (Aichert et al. 2012; De Wit et al. 2007; Kvam et al. 2021; Nguyen et al. 2018; Sánchez‐Kuhn et al. 2017; Vasconcelos et al. 2012). Our data recapitulate this pattern, with modest correlation coefficients between the outcome measures of the IST, Observe‐or‐Bet, TA, and MCQ on the one hand, and the BIS‐11 non‐planning and motor impulsivity scores on the other hand. Thus, our data highlight impulsivity as a set of behaviors mediated by overlapping cognitive, affective and neural mechanisms that can converge to produce potentially hazardous outcomes (Evenden 1999; Strickland and Johnson 2021).

In this context, we note that Masaki et al. (2016b) used the Cambridge Gambling Task to show that administration of ebselen to 20 participants moderated the early selection of bets from sequences of both increasing or decreasing stakes on probabilistic predictions, possibly reflecting strengthened control over motor‐based impulsivity captured by the stop‐signal response time on the SST (Kvam et al. 2021). By contrast here, we found no evidence that the stop‐signal response time was affected by ebselen administration in our larger sample, making it unlikely that the rapid action‐based impulsivity—potentially of considerable clinical significance in aggressive and self‐harming behaviors (Felthous et al. 2021; R. M. Jones et al. 2011; Sheard et al. 1976)—can be moderated directly by inhibition of IMPase and *myo*‐inositol activity.

Results from the TA discounting task and the MCQ represent only a modest departure from the general pattern above. Across three dependent measures from these tasks, ebselen was associated with reduced delay discounting scores among participants with low non‐planning impulsivity scores compared with placebo, but with higher discounting scores among participants with high BIS‐11 non‐planning subscale scores. This directly contradicts our secondary hypothesis that ebselen produces larger benefits in high‐trait compared with low‐trait impulsive individuals. Taken at face value, these findings even suggest that ebselen could be unhelpful in clinical groups presenting with maladaptive impulsive behaviors, by further strengthening preferences for immediate rewards over delayed but larger rewards. However, notwithstanding this possibility, it is more likely that these findings reflect sampling error that, in this experiment, placed more participants with both higher *k* (and lower AUC) values from the TA discounting task and MCQ as well as higher BIS‐11 non‐planning scores in the ebselen treatment group than in the placebo group.

In summary, the present findings throw some doubt on the hypothesis that impulse control—measured here as motor inhibition, the collection of adequate evidence before decisions to actions, and preferences for smaller sooner rewards compared with larger delayed rewards—is sensitive to modulation of *myo*‐inositol levels by doses of the IMPase inhibitor, ebselen.

## Author Contributions

All authors contributed to the study conception and design. Material preparation, data collection and analysis were performed by [Matthew P.G. John], [Timothy J. Davies], and [Robert D. Rogers]. The first draft of the manuscript was written by [Matthew P.G. John] and all authors commented on previous versions of the manuscript. All authors read and approved the final manuscript.

## Funding

This experiment was funded by an award from the Medical Research Council Development Pathway Funding Scheme (MR/L013150/1).

## Ethics Statement

The study was approved by the HRA and Health and Care Research Wales (HCRW) and given a favorable opinion by the National Health Service Research Ethics Committee (Wales REC 2) (IRAS ID: 244365). All participants provided voluntary and informed consent. The study was conducted in accordance with the World Medical Association's 1975 Declaration of Helsinki (as revised in 2013) and Bangor University's Research Ethics Policy.

## Conflicts of Interest

The authors are not in receipt of funding or support from pharmaceutical or any industry, commercial or charitable partners relevant to the research reported here. Two patents were filed on behalf of Oxford Innovation in relation to ebselen and impulsivity in clinical populations—US Patent App. 16/097, 239, 2019 and European Patent App. No. 17721810.4 between 2019 and 2025. These patents have been abandoned. None of the authors report any conflicts of interest.

## Supporting information


Supporting Information S1

## Data Availability

The data that support the findings of this study are openly available in “No moderation of impulsivity by myo‐inositol inhibition” at https://osf.io/45xu9/overview.
