# Supplementary material for: No Evidence of Moderated Impulsivity Following Administration of the IMPase Inhibitor Ebselen in Healthy Adults
Source: Hum Psychopharmacol. 2026 Apr 21;41:e70040. doi: 10.1002/hup.70040 (PMC13100343; doi:10.1002/hup.70040)
Supplement: Supplementary file 1 — Supporting Information S1 [file HUP-41-e70040-s001.docx]

**Methods**

| Dosing schedule of original 4-day design with ebselen, placebo and lithium. | | | |
| --- | --- | --- | --- |
|  | | | |
| Treatment Groups | | | |
|  | *Ebselen* | *Placebo* | *Lithium* |
| Day 1, 4pm | Placebo | Placebo | 740 mg |
| Day 2, 4pm | Placebo | Placebo | 1200 mg |
| Day 3, 9am | 600 mg | Placebo | Placebo |
| Day 3, 4pm | 600 mg | Placebo | 1200 mg |
| Day 4, 10am | 600 mg | Placebo | Placebo |
|  |  |  |  |
| Dosing schedule of amended 2-day design with ebselen and placebo | | | |
|  |  |  |  |
| Treatment Groups | | | |
|  | Ebselen | Placebo |  |
| Day 1, 9am | 600 mg | Placebo |  |
| Day 1, 4pm | 600 mg | Placebo |  |
| Day 2, 10am | 600 mg | Placebo |  |

*Table S1: Dosing schedules of the original between-groups 4-day, 3-arm protocol with ebselen, placebo and lithium and amended 2-arm protocol with ebselen and placebo.*

**Results**

| Measure | Effect | β | Test Statistic |
| --- | --- | --- | --- |
|  | | | |
| Information Sampling Task | | | |
| No. Boxes Opened | Treatment | 0.09 ± 2.20 | t(125) = 0.04, p = 0.97 |
|  | Design | -0.08 ± 1.85 | t(125) = -0.04, p = 0.96 |
|  | Treatment * Design | -0.52 ± 2.30 | t(125) = -0.22, p = 0.82 |
| Declaration Time | Treatment | 5.86 ± 4.71 | t(125) = 1.24, p = 0.22 |
|  | Design | 4.15 ± 3.98 | t(125) = 1.04, p = 0.30 |
|  | Treatment * Design | -5.25 ± 4.92 | t(125) = -1.07, p = 0.29 |
| No. Correct Declarations | Treatment | -0.13 ± 1.12 | t(125) = -0.11, p = 0.91 |
|  | Design | -0.7 ± 0.94 | t(125) = -0.74, p = 0.46 |
|  | Treatment * Design | 0.42 ± 1.17 | t(125) = 0.36, p = 0.72 |
| *p(Correct)* | Treatment | 0.02 ± 0.07 | t(125) = 0.29, p = 0.77 |
|  | Design | -0.03 ± 0.05 | t(125) = -0.49, p = 0.62 |
|  | Treatment * Design | -0.04 ± 0.07 | t(125) = -0.56, p = 0.58 |
| Observe-or-Bet | | | |
| Total Points | Treatment | -14.75 ± 18.34 | t(126) = -0.8, p = 0.42 |
|  | Design | -17.7 ± 15.47 | t(126) = -1.14, p = 0.25 |
|  | Treatment * Design | 16.48 ± 19.16 | t(126) = 0.86, p = 0.39 |
| Total Observation | Treatment | -17.75 ± 26.24 | t(126) = -0.68, p = 0.50 |
|  | Design | 11.33 ± 22.13 | t(126) = 0.51, p = 0.61 |
|  | Treatment * Design | 8.31 ± 27.41 | t(126) = 0.3, p = 0.76 |
|  | | | |
| Continuous Performance Test | | | |
| Correct Responses (Catch Trials) | Treatment | 1.37 ± 3.16 | t(122) = 0.44, p = 0.66 |
|  | Design | -0.04 ± 2.66 | t(122) = -0.02, p = 0.99 |
|  | Treatment * Design | -3.28 ± 3.3 | t(122) = -0.99, p = 0.32 |
| RTs (Catch Trials) | Treatment | 0.03 ± 0.02 | t(122) = 1.55, p = 0.12 |
|  | Design | 0.02 ± 0.02 | t(122) = 1.14, p = 0.26 |
|  | Treatment * Design | -0.04 ± 0.02 | t(122) = -1.85, p = 0.07 |
| Correct Responses (Target Trials) | Treatment | -3.75 ± 5.25 | t(122) = -0.71, p = 0.48 |
|  | Design | -5.53 ± 4.44 | t(122) = -1.25, p = 0.22 |
|  | Treatment * Design | 5.81 ± 5.5 | t(122) = 1.06, p = 0.29 |
| RTs (Target Trials) | Treatment | 0.02 ± 0.01 | t(122) = 1.12, p = 0.26 |
|  | Design | 0.01 ± 0.01 | t(122) = 0.68, p = 0.50 |
|  | Treatment * Design | -0.02 ± 0.02 | t(122) = -1.57, p = 0.12 |
| Commission Errors / Correct Detections | Treatment | 0.05 ± 0.17 | t(122) = 0.29, p = 0.77 |
|  | Design | 0.13 ± 0.14 | t(122) = 0.89, p = 0.38 |
|  | Treatment * Design | 0.01 ± 0.18 | t(122) = 0.05, p = 0.96 |
|  | | | |
| Titrating Alternatives | | | |
| *k* | Treatment | 0.20 ± 0.15 | t(124) = 1.32, p = 0.19 |
|  | Design | 0.10 ± 0.13 | t(124) = 0.8, p = 0.43 |
|  | Treatment * Design | -0.27 ± 0.16 | t(124) = -1.67, p = 0.10 |
| AUC | Treatment | 0.05 ± 0.09 | t(124) = 0.51, p = 0.61 |
|  | Design | -0.02 ± 0.08 | t(124) = -0.27, p = 0.79 |
|  | Treatment * Design | -0.03 ± 0.10 | t(124) = -0.35, p = 0.73 |
| Monetary Choice Questionnaire | | | |
| *k* | Treatment | -0.01 ± 0.02 | t(125) = -0.5, p = 0.62 |
|  | Design | -0.01 ± 0.02 | t(125) = -0.32, p = 0.75 |
|  | Treatment * Design | 0.001 ± 0.02 | t(125) = 0.1, p = 0.92 |

*Table S2: Comparison of treatment effects on principal outcome measures, controlling for design (2-day vs. 4-day). Note that all participants completing the SSRT task did so under the 2-day design.*

| **Measure** | **Effect** | **β** | **Statistical Significance** |
| --- | --- | --- | --- |
|  | | | |
| **Information Sampling Task** | | | |
| No. Boxes Opened | Treatment | 1.46 ± 2.43 | *t*(118) = 0.6, p = 0.55 |
|  | BIS (Nonplanning) | 0.01 ± 0.07 | *t*(118) = 0.2, p = 0.84 |
|  | Treatment * BIS (Nonplanning) | -0.08 ± 0.11 | *t*(118) = -0.72, p = 0.47 |
| *p*(Correct) | Treatment | 0.01 ± 0.07 | *t*(119) = 0.2, p = 0.85 |
|  | BIS (Nonplanning) | 0.001 ± 0.001 | *t*(119) = 0.07, p = 0.95 |
|  | Treatment * BIS (Nonplanning) | 0.001 ± 0.001 | *t*(119) = -0.12, p = 0.9 |
| **Observe-or-Best Task** | | | |
| Total Points | Treatment | 49.44 ± 26.19 | *t*(117) = 1.89, p = 0.06 |
|  | BIS (Nonplanning) | 0.05 ± 0.85 | *t*(117) = 0.06, p = 0.95 |
|  | Treatment * BIS (Nonplanning) | -2.23 ± 1.18 | *t*(117) = -1.89, p = 0.06 |
| Total Observations | Treatment | 29.21 ± 34.3 | *t*(120) = 0.85, p = 0.4 |
|  | BIS (Nonplanning) | 0.90 ± 1.06 | *t*(120) = 0.85, p = 0.4 |
|  | Treatment * BIS (Nonplanning) | -1.93 ± 1.51 | *t*(120) = -1.27, p = 0.2 |
| **Stop-Signal Task** | | | |
| *p*(Response \| Stop-Signal) | Treatment | 0.01 ± 0.05 | *t*(86) = 0.16, p = 0.87 |
|  | BIS | 0.001 ± 0.001 | *t*(86) = -0.03, p = 0.97 |
|  | Treatment * BIS (Motor) | 0.001 ± 0.001 | *t*(86) = -0.08, p = 0.93 |
| Stop-Signal Response Time | Treatment | -15.22 ± 54.63 | *t*(81) = -0.28, p = 0.78 |
|  | BIS | 0.37 ± 0.63 | *t*(81) = 0.59, p = 0.56 |
|  | Treatment * BIS (Motor) | 0.06 ± 0.92 | *t*(81) = 0.06, p = 0.95 |
| **Continuous Performance Test** | | | |
| Target Response Time (ms) | Treatment | -33.05 ± 24.07 | *t*(117) = -1.37, p = 0.17 |
|  | BIS | -0.30 ± 0.27 | *t*(117) = -1.11, p = 0.27 |
|  | Treatment * BIS (Motor) | 0.52 ± 0.40 | *t*(117) = 1.3, p = 0.2 |
| Commission Errors / Correct Detections | Treatment | 0.25 ± 0.22 | *t*(119) = 1.11, p = 0.27 |
|  | BIS | 0.001 ± 0.001 | *t*(119) = 1.54, p = 0.13 |
|  | Treatment * BIS (Motor) | 0.001 ± 0.001 | *t*(119) = -0.96, p = 0.34 |
| **Titrating Alternatives** | | | |
| Area Under the Curve | Treatment | 0.45 ± 0.25 | *t*(116) = 1.83, p = 0.07 |
|  | BIS | 0.001 ± 0.001 | *t*(116) = -0.38, p = 0.71 |
|  | Treatment * BIS (Nonplanning) | -0.01 ± 0.001 | *t*(116) = -1.87, p = 0.06 |
| Log *k* | Treatment | -2.11 ± 0.71 | *t*(116) = -2.97, p = 0.001 |
|  | BIS | 0.001 ± 0.01 | *t*(116) = -0.21, p = 0.83 |
|  | Treatment * BIS (Nonplanning) | 0.03 ± 0.01 | *t*(116) = 2.78, p = 0.01 |
| **Monetary Choice Questionnaire** | | | |
| Log *k* | Treatment | -1.99 ± 1.23 | *t*(118) = -1.61, p = 0.11 |
|  | BIS | 0.01 ± 0.01 | *t*(118) = 0.64, p = 0.53 |
|  | Treatment * BIS (Nonplanning) | 0.03 ± 0.02 | *t*(118) = 1.25, p = 0.21 |

*Table S3: Study results following removal of highly influential datapoints (Cook’s distances > 4 /* n*).*

| *Education Level* | *Ebselen* | *Placebo* | *Test Statistic* |
| --- | --- | --- | --- |
| Lower Secondary or Below | 4 | 2 | *χ*^2^(4) = 3.09, *p* = 0.54 |
| Upper Secondary | 25 | 29 |  |
| Post-Secondary Non-Tertiary | 9 | 4 |  |
| Bachelor's Degree | 16 | 17 |  |
| Postgraduate Degree | 10 | 12 |  |

*Table S4: Number of participants attaining each educational classification in the ebselen and placebo groups.*

| Symptom | Ebselen | Placebo | Test Statistic |
| --- | --- | --- | --- |
| Day 1 | | | |
| Headache | 60 / 4 / 2 / 0 | 51 / 10 / 2 / 1 | *χ*^2^(3) = 4.27, *p* = 0.23 |
| Stomach Upset | 61 / 4 / 0 / 1 | 60 / 4 / 0 / 0 | *χ*^2^(2) = 0.98, *p* = 0.61 |
| Nausea | 64 / 2 / 0 / 0 | 61 / 3 / 0 / 0 | *χ*^2^(1) = 0.01, *p* = 0.97 |
| Fatigue | 55 / 9 / 2 / 0 | 55 / 6 / 3 / 0 | *χ*^2^(2) = 0.77, *p* = 0.68 |
| Frequent Urination | 60 / 4 / 2 / 0 | 61 / 2 / 1 / 0 | *χ*^2^(2) = 0.98, *p* = 0.61 |
| Thirst | 57 / 7 / 2 / 0 | 57 / 6 / 1 / 0 | *χ*^2^(2) = 0.38, *p* = 0.83 |
| Dizziness | 65 / 1 / 0 / 0 | 60 / 4 / 0 / 0 | *χ*^2^(1) = 0.90, *p* = 0.34 |
| Day 2 | | | |
| Headache | 60 / 5 / 0 / 1 | 52 / 9 / 2 / 1 | *χ*^2^(3) = 3.68, *p* = 0.30 |
| Stomach Upset | 59 / 6 / 1 / 0 | 61 / 2 / 1 / 0 | *χ*^2^(2) = 2.00, *p* = 0.37 |
| Nausea | 65 / 0 / 1 / 0 | 62 / 1 / 1 / 0 | *χ*^2^(2) = 1.04, *p* = 0.59 |
| Fatigue | 60 / 3 / 3 / 0 | 52 / 8 / 4 / 0 | *χ*^2^(2) = 2.96, *p* = 0.23 |
| Frequent Urination | 63 / 2 / 1 / 0 | 58 / 3 / 3 / 0 | *χ*^2^(2) = 1.38, *p* = 0.50 |
| Thirst | 59 / 5 / 1 / 1 | 56 / 4 / 3 / 1 | *χ*^2^(3) = 1.16, *p* = 0.76 |
| Dizziness | 62 / 3 / 1 / 0 | 63 / 1 / 0 / 0 | *χ*^2^(2) = 1.98, *p* = 0.37 |

*Table S5: Number of participants reporting ‘No’ / ‘Mild’ / ‘Moderate’ / ‘Severe’ instances of each symptom on Day 1 and Day 2 (Day 3 and Day 4 in the 4-day design) in the ebselen and placebo groups.*

|  | Measure | Observed Value | Comparison Value |
| --- | --- | --- | --- |
| Information Sampling Task | Boxes Opened | 5.79±0.31 | 7.50±0.68  (Clark et al., 2006) |
|  | *p*(Correct) | 0.80±0.01 | 0.82±0.01  (Todesco et al., 2025) |
| Stop Signal Task | SSRT | 201±5.29ms | 241±3.51ms  (Brudan et al., 2024) |
|  | *p*(resp \| signal) | 0.47±0.01 | 0.50±0.001  (Verbruggen et al., 2013) |
| Continuous Performance Test | % Errors | 21.40±1.16 | 17.78±1.12  (Kirenskaya et al., 2021) |
| Titrating Alternatives Task | Log *k* | -1.69±0.06 | -1.63±0.13  (Bailey et al., 2018) |
|  | AUC | 0.34±0.02 | 0.39±0.01  (Halilova et al., 2024) |
| Monetary Choice Questionnaire | Log *k* | -4.69±0.40 | -3.72±0.19  (Boyd et al., 2024) |

*Table S6: Results of the IST, SST, CPT, Titrating Alternatives task, and MCQ, versus previous findings. Data are presented as mean ± 1 SE.*

| Measure | Effect | β | Test Statistic |
| --- | --- | --- | --- |
| AUC | Treatment | 0.35 ± 0.25 | *t*(116) = 1.38, *p* = 0.17 |
|  | BIS | -0.001 ± 0.001 | *t*(116) = -0.56, *p* = 0.58 |
|  | Treatment * BIS | -0.01 ± 0.001 | *t*(116) = -1.56, *p* = 0.12 |
| Log *k* | Treatment | -1.48 ± 0.68 | *t*(116) = -2.17, *p* = 0.03 |
|  | BIS | 0.001 ± 0.01 | *t*(116) = 0.39, *p* = 0.70 |
|  | Treatment * BIS | 0.03 ± 0.01 | *t*(116) = 2.26, *p* = 0.03 |

*Table S7: Results of the TA after removal of non-systematic discounters (Rung* et al*., 2018). Beta values are presented ± SE, with placebo treatment the reference level.*


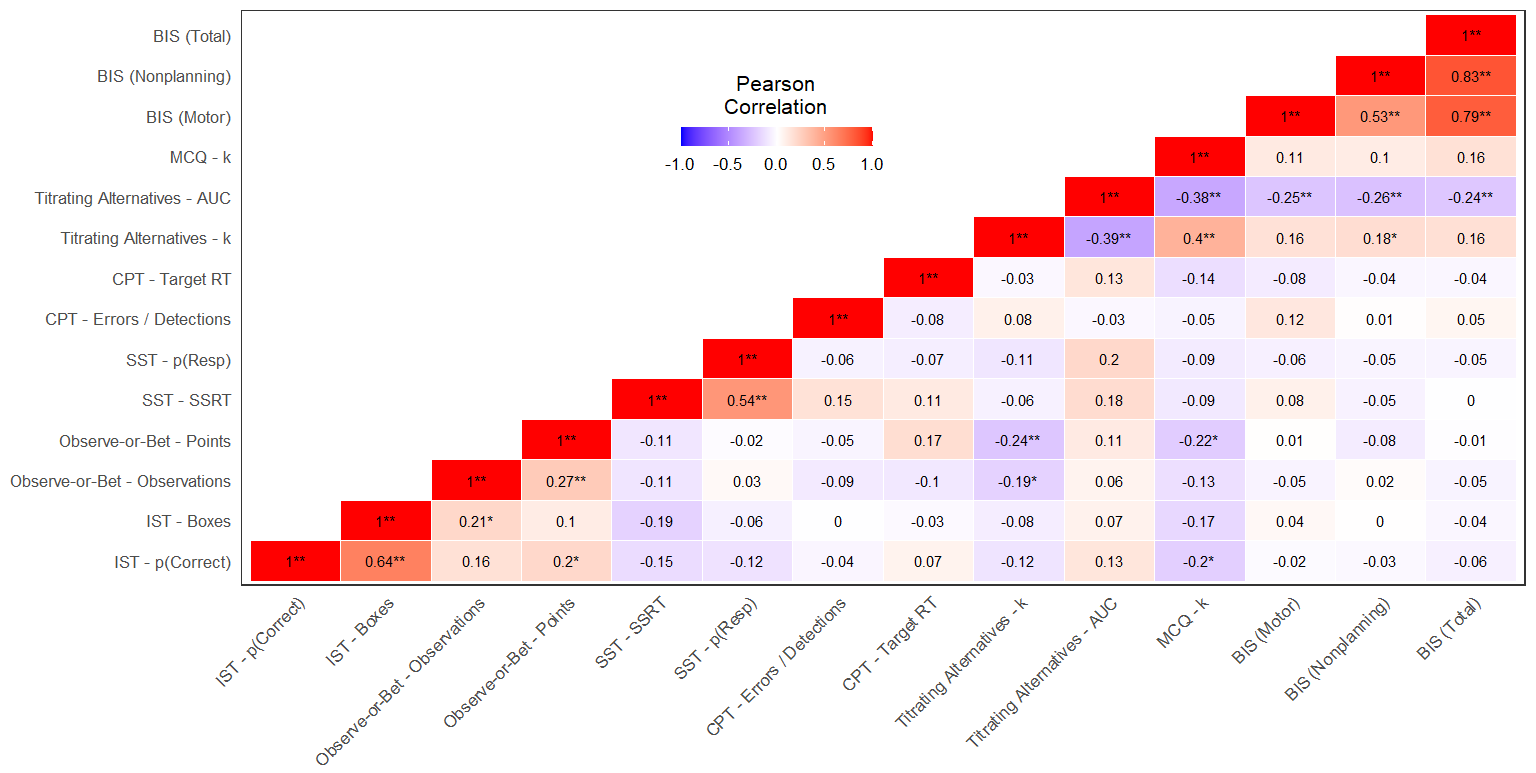


*Fig. S1: Inter- and intra-task correlations among primary outcome variables, pooled across treatment groups. Asterisks (*) indicate statistical significance at p* < 0.05, while double-asterisks (**) indicate significance at *p* < 0.01.


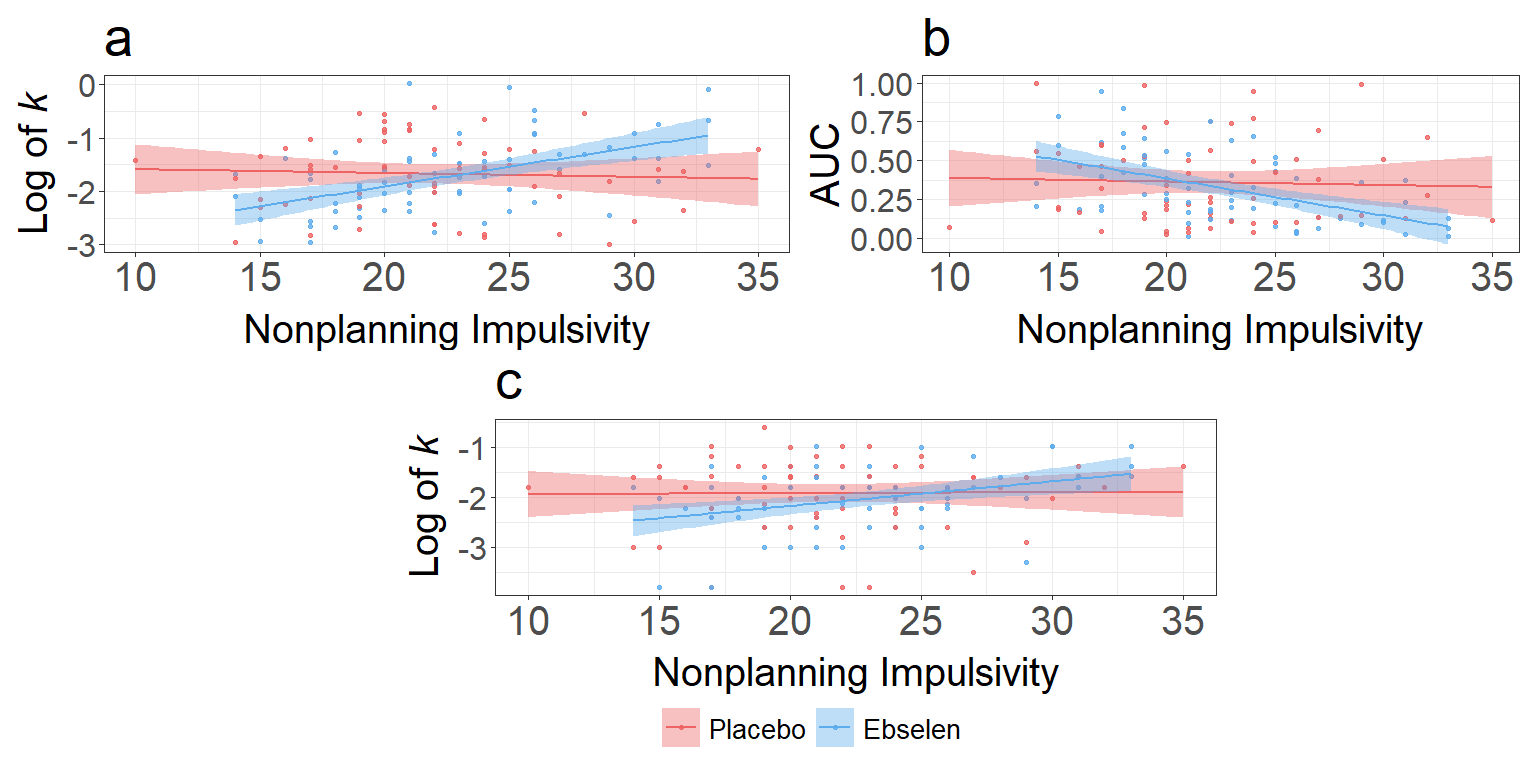


*Fig. S2 Among participants treated with ebselen, non-planning impulsivity was associated positively and negatively with k and AUC, respectively, in the Titrating Alternatives elicitation (a, b); and positively with* k *in the MCQ (c). For clarity,* k *is presented on the log scale.*

**References**

Bennett, D., Oldham, S., Dawson, A., Parkes, L., Murawski, C., & Yücel, M. (2017). Systematic overestimation of reflection impulsivity in the Information Sampling Task. *Biological Psychiatry*, *82* (*4*), 29-30.

Dougherty, D. M., Marsh, D. M., & Mathias, C. W. (2002). Immediate and delayed memory tasks: a computerized behavioural measure of memory, attention, and impulsivity. *Behaviour Research Methods, Instruments, & Computers*, *34* (*3*), 391-398.

Gray, J. C., Amlung, M. T., Palmer, A. A., & MacKillop, J. (2016). Syntax for calculation of discounting indices from the monetary choice questionnaire and probability discounting questionnaire. *Journal of the Experimental Analysis of Behaviour*, *106* (*2*), 156-163.

Kaplan, B. A., Amlung, M., Reed, D. D., Jarmolowicz, D. P., McKerchar, T. L., & Lemley, S. M. (2016). Automating scoring of delay discounting for the 21-and 27-item monetary choice questionnaires. *The Behaviour Analyst*, *39*, 293-304.

Kirby, K. N., Petry, N. M., & Bickel, W. K. (1999). Heroin addicts have higher discount rates for delayed rewards than non-drug-using controls. *Journal of Experimental Psychology: General*, *128* (*1*), 78-87.

Logan, G. D., & Cowan, W. B. (1984). On the ability to inhibit thought and action: A theory of an act of control. *Psychological Review*, *91* (*3*), 295-327.

Madden, G. J., Petry, N. M., & Johnson, P. S. (2009). Pathological gamblers discount probabilistic rewards less steeply than matched controls. *Experimental and Clinical Psychopharmacology*, *17* (*5*), 283-290.

Mazur, J. E. (1987). An adjusting procedure for studying delayed reinforcement. In M. L. Commons, J. E. Mazur, J. A. Nevin, & H. Rachlin (Eds). *Quantitative Analyses of Behaviour: Vol. 5. The Effect of Delay and of Intervening Events on Reinforcement Value* (pp. 55-73). Hillsdale, NJ: Erlbaum.

Navarro, D. J., Newell, B. R., & Schulze, C. (2016). Learning and choosing in an uncertain world: An investigation of the explore–exploit dilemma in static and dynamic environments. *Cognitive Psychology*, *85*, 43-77.

Peirce, J. W. (2007). PsychoPy: Psychophysics software in Python. *Journal of Neuroscience Methods*, *162* (*1-2*), 8-13.

Rachlin, H., Raineri, A., & Cross, D. (1991). Subjective probability and delay. *Journal of the Experimental Analysis of Behaviour*, *55* (*2*), 233-244.

Rogers, R. D., Tunbridge, E. M., Bhagwagar, Z., Drevets, W. C., Sahakian, B. J., & Carter, C. S. (2003). Tryptophan depletion alters the decision-making of healthy volunteers through altered processing of reward cues. *Neuropsychopharmacology*, *28* (*1*), 153-162.

Tversky, A., & Edwards, W. (1966). Information versus reward in binary choices. *Journal of Experimental Psychology*, *71* (*5*), 680-683.

Verbruggen, F., Adams, R., & Chambers, C. D. (2012). Proactive motor control reduces monetary risk taking in gambling. *Psychological Science*, *23* (*7*), 805-815.

Verbruggen, F., Chambers, C. D., & Logan, G. D. (2013). Fictitious inhibitory differences: how skewness and slowing distort the estimation of stopping latencies. *Psychological Science*, *24* (*3*), 352-362.

Verbruggen, F., & Logan, G. D. (2009). Models of response inhibition in the stop-signal and stop-change paradigms. *Neuroscience & Biobehavioral Reviews*, *33* (*5*), 647-661.
